# Extracellular Vesicles Loaded with Long Antisense RNAs Repress Severe Acute Respiratory Syndrome Coronavirus 2 Infection

**DOI:** 10.1089/nat.2023.0078

**Published:** 2024-06-17

**Authors:** Adi Idris, Surya Shrivastava, Aroon Supramaniam, Roslyn M. Ray, Galina Shevchenko, Dhruba Acharya, Nigel A.J. McMillan, Kevin V. Morris

**Affiliations:** ^1^ School of Pharmacy and Medical Science, Menzies Health Institute Queensland, Griffith University, Gold Coast Campus, Brisbane, Australia.; ^2^ Centre for Immunology and Infection Control, School of Biomedical Sciences, Queensland University of Technology, Brisbane, Australia.; ^3^ Center for Gene Therapy, City of Hope, Beckman Research Institute and Hematological Malignancy and Stem Cell Transplantation Institute at the City of Hope, Duarte, California, USA.; ^4^ Centre for Genomics and Personalised Health, School of Biomedical Sciences, Queensland University of Technology, Kelvin Grove, Brisbane, Australia.

**Keywords:** SARS-COV-2, antisense RNA, neural stem cell, exosome, extracellular vesicles

## Abstract

Long antisense RNAs (asRNAs) have been observed to repress HIV and other virus expression in a manner that is refractory to viral evolution. Severe acute respiratory syndrome coronavirus 2 (SARS-CoV-2), the causative agent of the coronavirus disease 2019 (COVID-19) disease, has a distinct ability to evolve resistance around antibody targeting, as was evident from the emergence of various SARS-CoV-2 spike antibody variants. Importantly, the effectiveness of current antivirals is waning due to the rapid emergence of new variants of concern, more recently the omicron variant. One means of avoiding the emergence of viral resistance is by using long asRNA to target SARS-CoV-2. Similar work has proven successful with HIV targeting by long asRNA. In this study, we describe a long asRNA targeting SARS-CoV-2 RNA-dependent RNA polymerase gene and the ability to deliver this RNA in extracellular vesicles (EVs) to repress virus expression. The observations presented in this study suggest that EV-delivered asRNAs are one means to targeting SARS-CoV-2 infection, which is both effective and broadly applicable as a means to control viral expression in the absence of mutation. This is the first demonstration of the use of engineered EVs to deliver long asRNA payloads for antiviral therapy.

## Introduction

Long antisense RNA (asRNA) targeting is one means to specifically repress transcript expression. Compared to small interfering RNAs (siRNA) and short antisense oligonucleotides (ASO), which range between 18 and 22 base pairs (bp) in length, long asRNAs are generally more than 200 bp long [1]. Although the exact mechanism is not well understood, asRNA-mediated gene repression can occur through transcriptional interference or sequestration [2], through the RNA interference machinery utilizing the ribonuclease III-like enzyme, Dicer [3], or recently, through the endogenous human adenosine deaminase acting on RNA enzyme [4].

Long asRNAs have been used previously to repress virus expression [5,6]. The effectiveness of current antivirals, including antibody-based ones, is waning due to the rapid emergence of new variants of concern (VOC), more recently the omicron variant [7]. Indeed, siRNAs [8–10] and short ASOs [11] have been used to repress severe acute respiratory syndrome coronavirus 2 (SARS-CoV-2) *in vivo.* The ability of the virus to evolve resistance around single siRNA targeting may, however, prove eventual, rendering this form of viral repression obsolete [12]. One means of avoiding the emergence of viral resistance to siRNA targeting is by using long asRNA to target SARS-CoV-2.

**FIG. 1.**
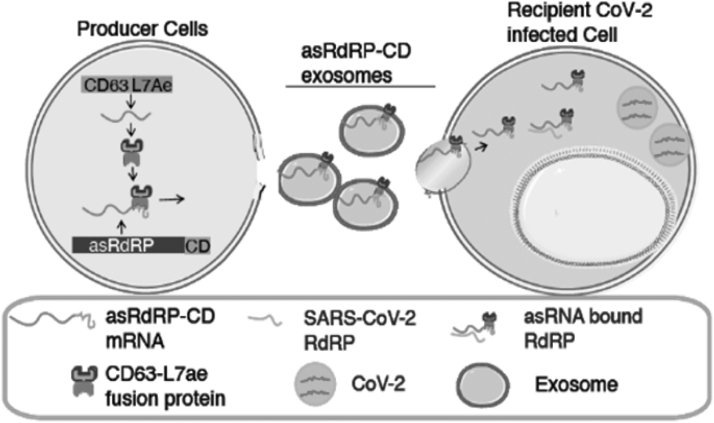
CD63-L7Ae and asRdRP-CD (anti-SARS-CoV-2 therapeutic RNA) are transfected as plasmids into A4-NSC producer cells. The CD63-L7Ae fusion protein binds to the RNA-binding C/D_box_ domain embedded into the therapeutic RNA (asRdRP-CD) and is packaged into the EVs. The EVs are then purified before exposing the EVs to target cells. asRdRP-CD, anti-SARS-CoV-2 therapeutic RNA targeting RdRP region; EVs, extracellular vesicles; NSC, neural stem cell; RdRP, RNA-dependent RNA polymerase; SARS-CoV-2, severe acute respiratory syndrome coronavirus 2.

A modular programmable therapeutic platform that can be developed to rapidly treat SARS-CoV-2 could prove paradigm shifting. In this study, we describe the use of extracellular vesicle (EV)-packaged anti-SARS-CoV-2 asRNAs to repress SARS-CoV-2 infection (Fig. 1). EVs are small nanoparticles (50–150 nm) that are constitutively shed by all cells and taken up by neighboring cells. EVs are the perfect delivery vehicle for various genetic therapeutics, including RNA, as they are relatively inert, nonimmunogenic, and anti-inflammatory [13,14]. We set out to use this system to package an asRNA targeting SARS-CoV-2, as asRNAs have been found to repress gene expression and given extensive target binding, the emergence of mutations against asRNA is rare [15].

## Materials and Methods

### Vectors

EXOsomal transfer into cell (EXOtic) device plasmids, pDB68 (Connexin43 S368A), pDB30 (CD63-nLuc), pSA465 (CD63-L7Ae), and pSA462 (nanoluc-C/D_box_), were also kind gifts from Dr. Martin Fussenegger [16]. The C/D_box_ was introduced in the 3′’UTR of asRNAs through standard oligo cloning methods. asRNAs were all cloned into a pcDNA3.1(+) mammalian expression vector.

### Cell lines

HEK293T cells, Vero E6 cells, immortalized mesenchymal stem cells (MSC), lung derived-induced pluripotent stem cells, and neural stem cells (NSCs) (A4 lineage) were cultured in Dulbecco's modified Eagle's medium (DMEM) (Thermo Fisher Scientific) supplemented with 10% fetal bovine serum (FBS) and incubated at 37°C and 5% CO^2^. Monocyte-derived macrophages (MDMs) were cultured in RPMI1640 under similar conditions.

### asRNA design

Long asRNA sequences were designed toward regions of the SARS-CoV-2 viral genome, which were functionally important and have a high degree of sequence conservation between the SARS-CoV-1 and SARS-CoV-2 genomes, including current circulating SARS-CoV-2 VOCs. These regions include the SARS-CoV-2: (1) Nucleoprotein (N) genomic region that plays a critical role in virion assembly; (2) 5′ region of the RNA-dependent RNA polymerase (RdRP) (*nsp12*) gene that includes the frameshift stimulating pseudoknot, and (3) catalytic region (Cat) of the RdRP (*nsp12*) gene, which is critical for the replication of the viral genome inside othe host cell. For each of these regions, the length varied between 600 and 800 bp. As a control, an asRNA designed toward enhanced green fluorescent protein (eGFP) was used. Each of the sequences used is listed in Supplementary Table S1.

### asRNA cloning and transfection *in vitro*

Long asRNAs used in this study were cloned in a manner to be expressed from the cytomegalovirus promoter. asRNAs were cloned into a pcDNA3.1(+) plasmid. Nontargeting asRNAs are against eGFP. Cells were seeded overnight to 70%–80% confluency before transfecting asRNA plasmids with FuGENE 6 (Promega) in OptiMEM (Gibco-Invitrogen), as per the manufacturer's protocol.

### Virus

SARS-CoV-2 Wuhan (Ancestral—VIC1) strain was obtained from the Peter Doherty Institute for Infection and Immunity and Melbourne Health, Victoria, Australia, and cultured in Vero E6 cells.

### Viral plaque and immunoplaque assays

For viral plaque assays, Vero E6 cells were infected with SARS-CoV-2 for 1 h before overlaying with 1% methylcellulose viscosity (4,000 centipoises) (Sigma-Aldrich). Cells were incubated for 4 days at 37°C before fixing in 8% formaldehyde and stained with 1% crystal violet to visualize plaques. Viral immunoplaque assays for SARS-CoV-2 were performed on Vero E6 cells, as described previously [9], using recombinant monoclonal antibodies that recognize SARS-CoV-2.

### Viral copy number determination

To determine viral copy numbers in infected cells, digital polymerase chain reaction (PCR) against the N gene of SARS-CoV-2 (CDC primers from IDT—SARS-CoV-2 N1) was performed in Quant-Studio 3D Digital PCR 20K chips (Thermo Scientific) on a ProFlex 2 × Flat Block Thermal Cycler (Thermo Scientific). Results are analyzed on the QuantStudio 3D Analysis Suite software (Thermo Scientific) and expressed as viral copies per microliter of template RNA.

### EV production, asRNA packaging, and characterization

Control and asRNA-packaged EVs in this study were produced using the following plasmids from the EXOtic packaging system (Table 1):

**Table 1.** EXOtic Packaging System Plasmids

DB30	pDB30 (CD63-nLuc)+pDB68 (connexion EV release)
asRdRP Cat 800	pSA465 (CD63-L7Ae)+pcDNA3-as-RdRP800 Cat-CD+pDB68 (connexion EV release)
asControl	pSA465 (CD63-L7Ae)+pcDNA3-as-GFP-CD+pDB68 (connexion EV release)

EXOtic, EXOsomal transfer into cells.

Producer cells were transfected using Lipofectamine 3000 (Thermo Fisher Scientific), according to the manufacturer's instructions, over 24 h before washing cells with DMEM and replacing media with DMEM +10% EV-depleted FBS (Thermo Fisher Scientific). Supernatant was collected at 48 and 96 h later and centrifuged at 300 *g* for 10 min at 4°C. The viability of cells was determined at the time of EV collection, which was greater than 90%. Supernatant was transferred to a new 50-mL conical tube and further centrifuged at 2,000 *g* for 20 min at 4°C before passing the supernatant through a 0.45-mm filter (Millipore).

**FIG. 2.**
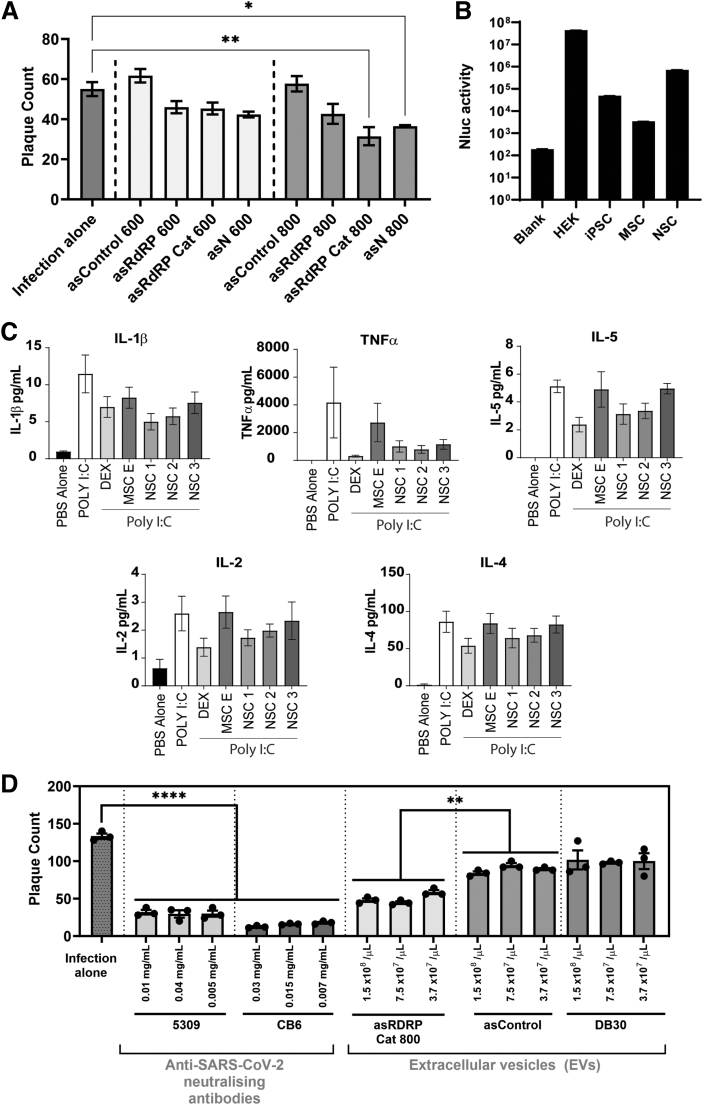
Delivery of asRNAs targeting the SARS-CoV-2 RdRP gene packaged in NSC EVs represses SARS-CoV-2 infection *in vitro*. **(A)** Vero E6 cells in 96-well plates were transfected with 0.1 μg of indicated plasmids with FuGENE 6 for 24 h before infecting with 62.5 PFUs of live SARS-CoV-2 virus (ancestral) for a further 24 h before enumerating viral plaques by the immunoplaque assay. The test asRNAs used in this study target regions within the SARS-CoV-2 nucleoprotein (N) genomic region (asN), the 5′ region of the RdRP (nsp12) gene that includes the frameshift stimulating pseudoknot (asRdRP), and the catalytic region (Cat) of the RdRP (nsp12) gene (asRdRP Cat). For each of these regions, the length varied between 600 and 800 bp (designated as suffixes, 600 or 800). Control asRNA against eGFP of varying sizes is used as a nontargeting asRNA. Data are representative of one out of three independent experiments. Triplicate treated cells are shown with the standard error of the mean of triplicate treatments and asterisks represent level of statistical significance where *P*-values of <0.05 (*) and <0.01 (**) were considered statistically significant, as determined by a one-way ANOVA test. **(B)** The relative packaging efficiency of CD63-NLuc in isolated and purified EVs from various cell systems (HEK293T, lung-derived iPSC, MSC, and NSC) previously transfected with pDB30 (CD63-nLuc) plasmid for 24 h was determined from 1 × 10^6^ EVs. Blank denotes ultracentrifuged supernatant from untransfected cells (ie, no EVs). Luminescence (Nanoluc activity) was measured on a luminometer (Glomax explorer). Bars represent an average of three technical replicates ± SEM. **(C)** GM-CSF-primed human MDMs were untreated (PBS alone) or treated with poly I:C alone or in combination with either DEX, MSC-derived EVs (MSC E; 10^8^ EVs/well) or NSC-derived EVs at three different concentrations (1: 10^7^ EVs/well; 2: 10^8^ EVs/well, and 3: 10^9^ EVs/well) for 24 h. Thereafter, supernatants were harvested, clarified, and processed for cytokine detection on a Bio-Plex^®^ 200. Analytes shown are IL-1β, TNF-α, IL-2, IL-5, and IL-4. **(D)** EVs or neutralizing antibodies were used neat or serially diluted twofold before mixing with 250 PFU of live SARS-CoV-2 (ancestral) for 30 min at RT before infecting Vero E6 cells for 1 h at 37°C. Virus and neutralizing antibody/EV mixtures were then removed before performing a viral plaque assay for 5 days. asControl is a nontargeting asRNA against eGFP and DB30 is an empty vector control EV containing only CD63 and Nluc. Two clones of neutralizing antibodies against SARS-CoV-2 were used as a positive control. Triplicate treated cells are shown with the standard error of the mean of triplicate treatments and asterisks represent level of statistical significance where *P*-values of <0.01 (**) and <0.001 (****) were considered statistically significant, as determined by a one-way ANOVA test. asRNA, antisense RNAs; DEX, dexamethasone; GM-CSF, granulocyte-macrophage colony-stimulating factor; IL, interleukin; iPSC, induced pluripotent stem cells; MDMs, monocytes-derived macrophages; MSC, mesenchymal stem cells; NSC, neural stem cells; PBS, phosphate-buffered saline; PFU, plaque-forming unit; SEM, standard error of mean; TNF, tumor necrosis factor.

The filtered supernatant was then centrifuged at 100,000 *g* for 120 min at 4°C to pellet the EVs, which were resuspended in phosphate-buffered saline (PBS). The final precipitate was resuspended in sterile PBS and passed through 0.22-micron Ultrafree^®^ Centrifugal Filter Units (Millipore Sigma). EVs were then quantified using Nanoparticle Tracking Analysis on a NanoSight (Malvern Panalytical) and further confirmed that these are CD63-positive EVs by determining luciferase activity using the Nluc tagged to CD63 relative to ultracentrifuged supernatant from untransfected cells (ie, no EVs) (Fig. 2B). For all *in vitro* experiments, exosome to cell ratio of 10^4^ exosomes per cell was maintained.

### Determining luciferase activity in EVs

Nano-Glo^®^ Luciferase assay system (Promega) was used to determine the luciferase activity in purified nanoluc (Nluc) exosomes as per the manufacturer's instructions before measuring luciferase activity on the Promega GloMax Discover Microplate Reader Detection System with GM3000 Software (Promega).

### Efficiency of asRNA packaging into EVs

The packaging efficiency of 800 bp long asRNA overlapping the RdRP gene (asRdRP 800) in CD63-L7ae and CD63 EVs from HEK293T cells was determined by co-transfecting 50 ng CD63-L7ae with 50 ng asRdRP. Twenty four hours later, the supernatant was collected and passed through a filter column for RNA extraction detected asRdRP 800 RNA by real-time PCR (RT-PCR) relative to U6sRNA (housekeeping gene).

### RT-PCR detection of asRNAs in EVs

RNA was isolated from exosomes using the Maxwell^®^ automated simplyRNA isolation kit according to the manufacturer's instructions (Promega). Equal amounts of RNA were used for Luna^®^ Universal One-Step RT-qPCR Kit (NEB) according to the manufacturer's instruction. Roche^®^ LightCycler 96 was used to perform the RT-qPCR and results were analyzed using the LightCycler 96 software (Roche).

### EV biodistribution in mice

NSC EVs diluted in sterile PBS were complexed with DiIC18(7);1,1′-dioctadecyl-3,3,3′,3′-tetramethylindotricarbocyanine iodide (DIR), a lipophilic, near-infrared fluorescent cyanine dye at a final concentration of 1.55 ng/μL DIR. A total of 10^8^ NSC EVs were administered to BALB/c mice intravenously (IV) by tail vein injection (20 μL). Fluorescence was detected using a single photon animal imager at various indicated time points post-administration.

### Cytokine profiling and liver enzyme analysis of mouse administered with EVs

A total of 100 μL of 100 billion HEK derived EVs per mouse (C57BL/6) were injected through IV, or 100 μL of 30 billion NSC-derived EVs were injected per mouse through IV. PBS was used as control. Mice bled at 4 and 72 h postinjection and serum was collected to measure cytokine expression by performing RT-PCR of mouse interferon (IFN)α/β mRNA. Two hundred microliters of blood was taken from mice for VetScan Mammalian Liver Profile analysis (Abaxis) 72 h post-EV administration. Liver enzymes analyzed were alkaline phosphatase, aspartate aminotransferase, and blood urea nitrogen. For SARS-CoV-2-infected mice lungs, tissues were homogenized in trizol before extracting RNA for RT-PCR analysis to determine mouse IFNβ mRNA expression on the Rotor-Gene (Thermo Fisher Scientific) using the Rotor-Gene SYBR Green RT-PCR kit (Qiagen, Hilden, Germany).

### Cytokine detection from human macrophages

Blood from consented and deidentified donors was used in this study under an approved IRB 19582 (City of Hope, Duarte, CA). Human monocyte isolation and macrophage differentiation were performed as previously described [17]. Granulocyte-macrophage colony-stimulating factor (GM-CSF) primed human macrophages were either untreated (sterile PBS) or treated with polyinosinic:polycytidylic (poly I:C; Sigma Aldrich), dexamethasone (Sigma Aldrich), MSC EVs, or increasing concentrations of NSC EVs, and supernatant collected 24 h later. Supernatants collected from cells were subjected to cytokine detection by Luminex using a Cytokine 10-Plex Human Panels (LHC0001M; Thermo Fisher Scientific) on a Bio-Plex^®^ 200 (Bio-Rad) by the Analytical Pharmacology Core (City of Hope).

### SARS-CoV-2 *in vivo* work

K18-hACE2 mice (3–4 months old) were purchased from the Jackson Laboratory (Bar Harbor, ME) and bred in-house at the Griffith University Animal Resource Center. Mice were intranasally (IN) infected with 10^4^ plaque-forming unit (PFU) (20 μL total volume) of live SARS-CoV-2, while under isoflurane anesthesia. Mice were subsequently treated with EVs retro-orbitally (IV) (100 μL total volume), while under isoflurane anesthesia. Mice were monitored daily for weighing and clinical scoring.

## Results and Discussion

To determine if SARS-CoV-2 is susceptible to asRNA-directed silencing, we generated and tested various asRNAs of various lengths to highly conserved regions within the RdRP and nucleoprotein (N) genes of SARS-CoV-2 (Supplementary Fig. S1). The N protein plays a critical role in virion assembly, whereas RdRP (*nsp12* gene) is critical for the replication of the viral genome inside the host cell. We found that an 800 bp long asRNA overlapping the RdRP catalytic (Cat) region (asRdRP Cat 800) reproducibly repressed SARS-CoV-2 infection with two independent asRNA controls (Fig. 2A and >Supplementary Fig. S2A) and confirmed by knockdown of SARS-CoV-2 viral copy numbers (Supplementary Fig. S2B).

Next, we designed the top candidate asRNA, asRdRP Cat 800, to be packaged with the EXOtic system into EVs. The EXOtic EV packaging system allows packaging of virtually any RNA into any cell type [16]. As we have previously generated EVs from HEK293T cells [18,19], we wanted to compare the EXOtic packaging efficiency of nanoluciferase (Nluc) in EVs purified from other cell types, in particular, stem cells relative to HEK293T cells. We found that NSCs efficiently package Nluc in EVs (Fig. 2B). Using the EXOtic system, we then proceeded to package asRdRP Cat 800 into NSC EVs.

In this system, the ubiquitous exosome marker protein CD63, a tetraspanin protein, is fused to an archaebacterial derived L7Ae peptide, which allows for the recruitment and encapsulation of those RNA containing C/D_box_ to budding exosomes. The DNA sequence for C/D_box_ was cloned at the 3′end of asRdRP Cat 800 to express mRNA containing the C/D_box_ RNA domain. Importantly, we found that the addition of a C/Dbox RNA domain into asRdRP Cat 800 allowed more effective asRdRP Cat 800 packaging into EVs (Supplementary Fig. S3). These data suggest that asRdRP Cat 800 is a viable RNA capable of repressing SARS-CoV-2 infection *in vitro* and that this antisense transcript can be packaged into EVs, similar to observations with protein coding mRNAs [19,20].

An added benefit from using NSCs to produce our antiviral EVs is that NSCs have been observed to impart anti-inflammatory effects [21], suggesting the use of these cells to make EVs may provide an approach that not only delivers antiviral RNAs to virus infected cells but may also diminish SARS-CoV-2-mediated immune dysregulation and severe acute respiratory distress. To determine if the NSCs used to develop the asRdRP Cat 800 containing EVs are imbued with anti-inflammatory properties, we treated polyinosinic:polycytidylic acid (poly I:C)-exposed GM-CSF primed human MDMs with NSC- and MSC-derived EVs, which have been reported to have anti-inflammatory properties [22,23].

We found that NSCs reduced key cytokines [interleukin (IL)-1β, tumor necrosis factor (TNF)-α, IL-2, IL-5, and IL-4] that drive coronavirus disease 2019 (COVID-19) severe acute respiratory distress complications [24] to similar levels in poly I:C-exposed cells treated with a known anti-inflammatory agent, dexamethasone (Fig. 2C). We also demonstrated that NSC-derived EVs comparably had better anti-inflammatory effects than MSC-derived EVs. Overall, this shows that NSC-derived EVs can offer an added anti-inflammatory effect when used to deliver the antiviral RNA payloads to target cells. We then tested the ability of asRdRP Cat 800 packaged in NSC EVs to dampen SARS-CoV-2 infection *in vitro*. Compared to a nontargeting control asRNA, asRdRP Cat 800 EVs significantly repressed SARS-CoV-2 replication (Fig. 2D).

**FIG. 3.**
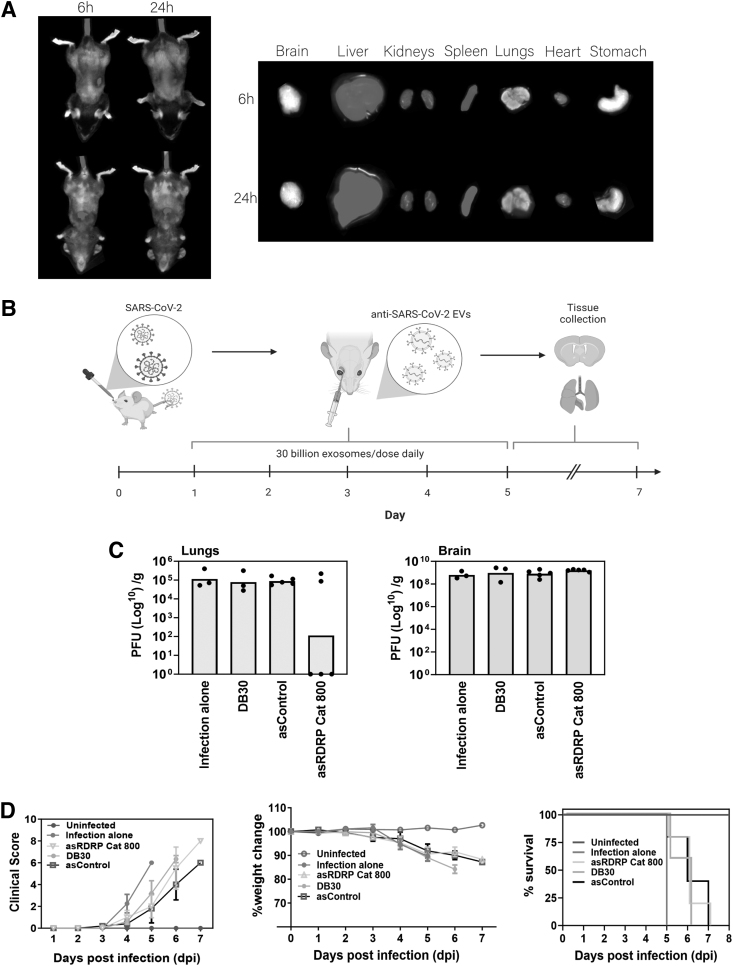
Delivery of NSC EVs containing anti-SARS-CoV-2 asRNA reduces viral lung infection in mice. **(A)** 10^8^ NSC EVs complexed with DIR were administered to BALB/c mice IV by tail vein injection (20 μL). Fluorescence was detected using a single-photon animal imager at indicated various time points post-administration. **(B)** Seven- to thirteen-week-old K18-hACE2 mice were intranasally infected with 10^4^ PFU of SARS-CoV-2. Mice were IV administered with 30 billion NSC EVs in 100 μL of PBS by retro-orbital injection at 1, 2, 3, 4, and 5 dpi. At 5–7 dpi, mice were euthanized, and lung and brain tissues were harvested and homogenized for viral immunoplaque assays. asControl are EVs containing a nontargeting asRNA against GFP and DB30 is an empty vector control EV containing only CD63 and Nluc. **(C)** The amount of infectious virus particles in lung and brain tissues at 5–7 dpi (*n* = 3–5 mice) was determined by viral immunoplaque assays on Vero E6 cells and expressed as PFU per gram (g) of tissue. Each data point represents one mouse and bars represent the geometric mean. No statistical significance was found when a one-way ANOVA was performed between virus alone and other treatment groups. **(D)** Mice were weighed and scored daily until the experimental endpoint for disease progression. Body weight (weight change), the clinical score, and probability of survival were evaluated at the indicated dpi. Mice that lost >15% of their initial body weight were humanely euthanized and plotted as a nonsurvivor. The clinical score was evaluated based on locomotion, behavior, and appearance. Each data point represents the average ± SEM of 3–5 mice. No statistical significance was found when a two-way ANOVA was performed between infection alone and other treatment groups (excluding uninfected). DIR, dioctadecyl-3,3,3′,3′-tetramethylindotricarbocyanine iodide; dpi, days post-infection; GFP, green fluorescent protein; IV, intravenously.

To explore the ability of these EVs to functionally inhibit CoV-2 *in vivo*, we explored the biodistribution of NSC EVs *in vivo* following IV administration (IV). We find that the Nluc containing NSC EVs disseminate to liver, spleen, and lungs when IV delivered (Fig. 3A). IV delivery may prove advantageous in localizing delivery to the lung, an organ afflicted by SARS-CoV-2. To assess this notion, we tested the EVs in a SARS-CoV-2 mouse model by testing the therapeutic effect of delivering five daily doses of asRdRP Cat 800 EVs by IV administration (Fig. 3B).

We also found that 30 billion NSC EVs per IV injection was well tolerated with no observable change in liver enzyme function (Supplementary Fig. S4) and was not immunostimulatory (Supplementary Fig. S5), suggesting that these EVs are safe to be delivered into mice. Hence, we used this amount of NSC EVs for the subsequent *in vivo* study. Lungs from three out of five mice treated with asRdRP Cat 800 EVs had no detectable SARS-CoV-2 infection when compared to its control, asGFP packaged in EVs (asControl) (Fig. 3C), suggesting that asRdRP Cat 800 EVs are able to exert a notable antiviral effect in the lungs of mice, although not in all the treated mice. We reasoned that this incomplete antiviral activity could either be attributed to poor EV biodistribution in the lungs (Fig. 3A) or be limited by the amount of daily EVs administered (ie, 30 billion EVs).

Indeed, work by Shrivastava *et al.* [19] showed that therapeutic daily IV delivery of 100 billion EVs repressed HIV-I viral load in organs as far as the brain. Future work will focus on delivering higher doses of EVs either IN or intratracheally as EVs will directly reach the lungs in higher amounts compared to IV delivered EVs [25]. Importantly, intranasal administration of NSC [26]- and astrocyte cell [27]-derived EVs has been shown to deliver EVs to the brain. Indeed, we failed to see any antiviral activity with asRdRP Cat 800 EVs in the brains of mice when delivered by IV (Fig. 3A). Within the context of clinical long COVID caused by SARS-CoV-2 [28], a therapeutic EV approach that can deliver anti-SARS-CoV-2 asRNAs into the brain would be clinically beneficial.

Although not statistically significant, we observed that all EV-treated SARS-CoV-2-infected mice have improved clinical well-being and survival (Fig. 3D). This clinical improvement could possibly be attributed to the anti-inflammatory capacity of these NSC EVs (Fig. 2C). Although we showed that these NSC EVs did not activate type I IFNs in either serum from uninfected mice (Supplementary Fig. S5) or lungs from SARS-CoV-2-infected mice (Supplementary Fig. S6), further work is still needed to conclusively determine that these EVs are indeed exerting an anti-inflammatory effect in infected mice by performing a detailed cytokine panel analysis in all organs.

There are several other limitations in this study. SARS-CoV-2 variant used in this study is the ancestral variant, not the most recent VOC. However, asRdRP Cat 800 was designed within a highly conserved region of the RdRP gene, where these is >90% conservation between SARS-CoV-1 and SARS-CoV-2, including current SARS-CoV-2 VOCs (Supplementary Fig. S1). Indeed, the RdRP region (*nsp12*) that asRdRP Cat 800 targets is highly conserved across all SARS-CoV-2 VOCs [29], making this gene highly druggable for antiviral targeting. Therefore, the antiviral activity of our asRNAs is expected to be equipotent across all SARS-CoV-2 VOCs.

Future work aims to demonstrate this conclusively in other SARS-CoV-2 VOCs, both *in vitro* and *in vivo*. To our knowledge, this is the first demonstration of the use of engineered EVs to deliver long asRNA payloads for antiviral therapy. Overall, our pre-clinical data demonstrate that anti-SARS-CoV-2 asRNAs delivered in EVs can ameliorate lung viral infection. Collectively, these data suggest that EV delivery of RNA therapeutics may prove to be a viable approach as an antiviral agent. Our data provide a proof-of-concept approach that this scalable and cost-effective platform technology can be easily amended to target novel pandemic RNA viruses beyond SARS-CoV-2.

### Ethics statement

All animal experiments were performed in compliance with relevant laws and institutional guidelines and in accordance with the ethical standards of the Declaration of Helsinki. All animal care and procedures have been performed according to protocols reviewed and approved by the City of Hope Institutional Animal Care and Use Committee (IACUC) held by the principal investigator for this application (Kevin Morris, IACUC 16095). All SARS-CoV-2 animal work was conducted in a BSL3-approved animal facility at Griffith University (Animal ethics approval: MHIQ/09/20/AEC). Blood from consented and deidentified donors was used in this study under an approved IRB 19582 (City of Hope).

## Data Availability Statement

All data generated or analyzed during this study are included in this article.
