# Supplementary material for: Extracellular Vesicles Loaded with Long Antisense RNAs Repress Severe Acute Respiratory Syndrome Coronavirus 2 Infection
Source: Nucleic Acid Ther. 2024 Jun 17;34(3):101–8. doi: 10.1089/nat.2023.0078 (PMC11296208; doi:10.1089/nat.2023.0078)

**Figure S1** Long asRNA sequences were designed towards regions of the SARS-CoV-2 viral genome that were functionally important and have a high degree of sequence conservation between the SARS-CoV-1 and SARS-CoV-2 genomes, including current SARS-CoV-2 VOCs.


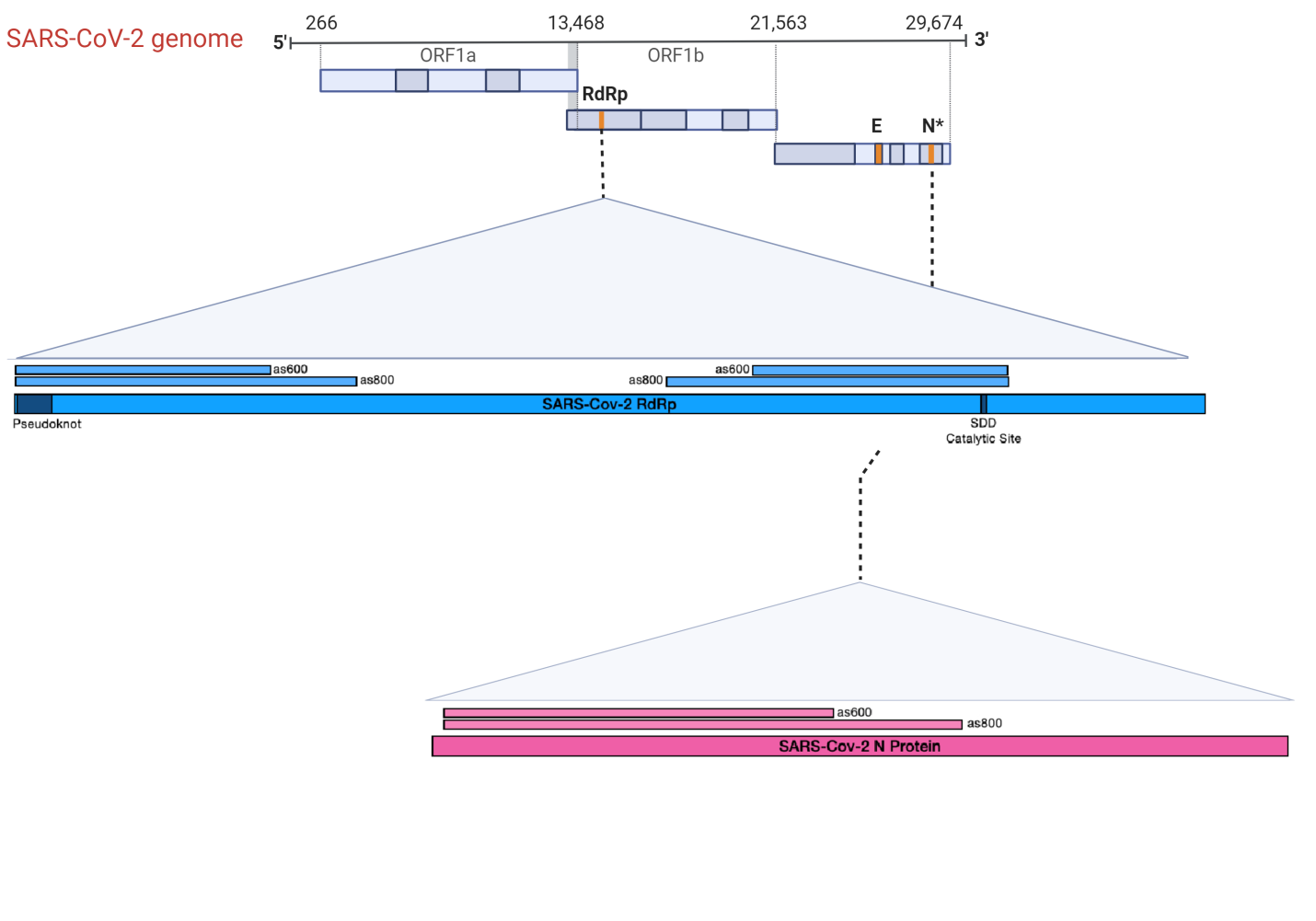

Supplement: Supplementary Figure S1 [file nat.2023.0078_suppl_figures1.docx]
