# Supplementary material for: Extracellular Vesicles Loaded with Long Antisense RNAs Repress Severe Acute Respiratory Syndrome Coronavirus 2 Infection
Source: Nucleic Acid Ther. 2024 Jun 17;34(3):101–8. doi: 10.1089/nat.2023.0078 (PMC11296208; doi:10.1089/nat.2023.0078)

**Figure S2**

1. Vero E6 cells in 96-well plates were transfected with 0.1μg of indicated plasmids with FuGENE 6 for 24h before infecting with 62.5 plaque forming units (PFU) of live SARS-CoV-2 virus (ancestral) for a further 24h before enumerating viral plaques by the immunoplaque assay. Control asRNA against HIV-1 reverse transcriptase (RT) is used as a non-targeting asRNA. Data is representative of one out of two independent experiments. Triplicate treated cells are shown with the standard error of the mean of triplicate treatments and asterisks represents level of statistical significance where p values of <0.05 (*) and <0.01 (**) were considered statistically significant as determined by a One-way ANOVA test.
2. Vero E6 cells in 96-well plates were transfected with 0.1µg of indicated plasmids with FuGENE 6 for 24h before infecting with 62.5 plaque forming units (PFU) of live SARS-CoV-2 virus (ancestral) for a further 24h before viral copy numbers were determined by digital droplet PCR against SARS-CoV-2 N gene. Control asRNA against eGFP was used as a non-targeting asRNA. Bars represent the mean viral copy numbers/ µl and error bars represent the SEM of triplicate treatments.


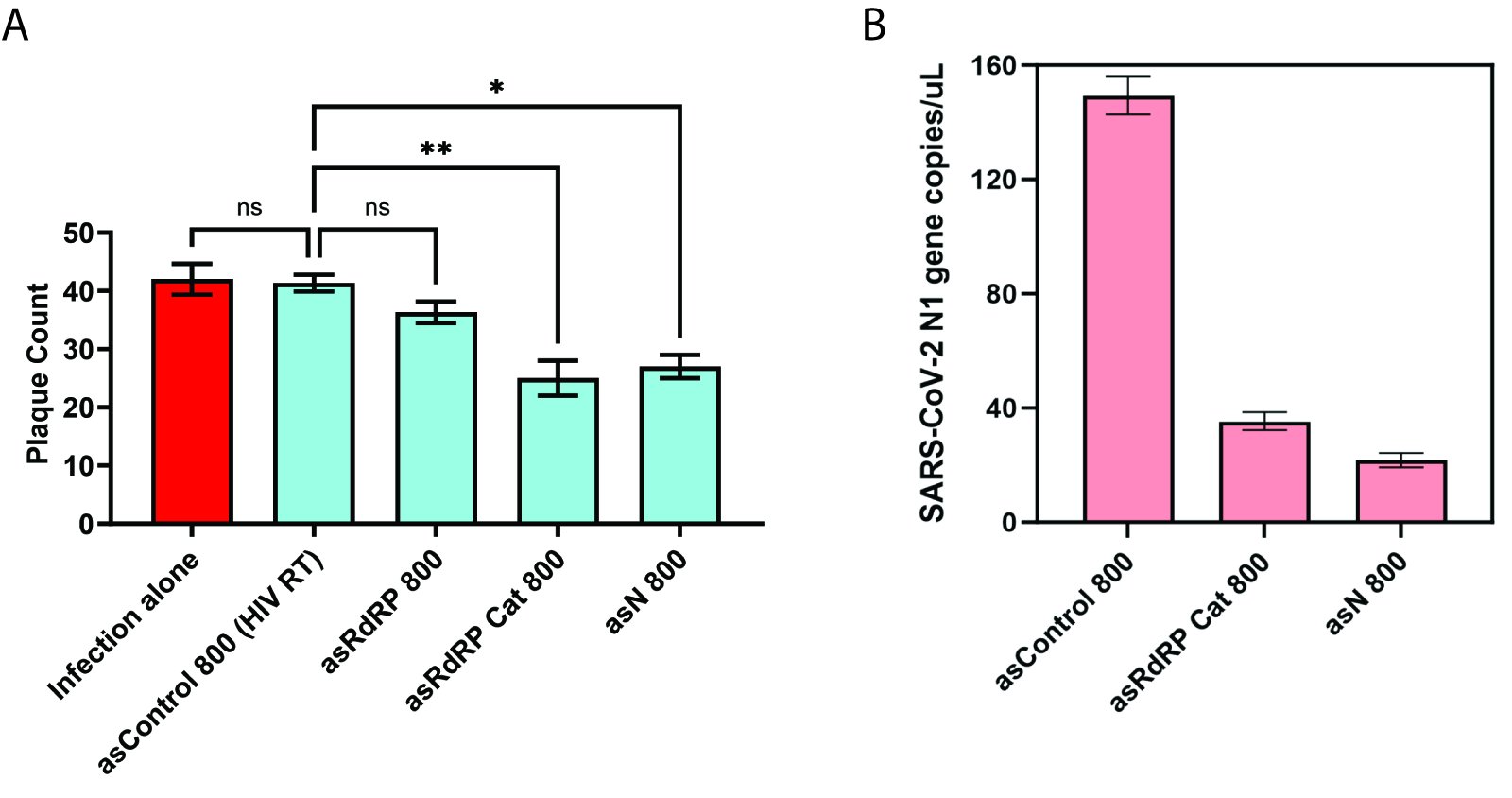

Supplement: Supplementary Figure S2 [file nat.2023.0078_suppl_figures2.docx]
