# Supplementary material for: Extracellular Vesicles Loaded with Long Antisense RNAs Repress Severe Acute Respiratory Syndrome Coronavirus 2 Infection
Source: Nucleic Acid Ther. 2024 Jun 17;34(3):101–8. doi: 10.1089/nat.2023.0078 (PMC11296208; doi:10.1089/nat.2023.0078)

**Figure S3** Packaging efficiency of as800bp asRNA alone (-CD) or containing a C/D_box_ RNA domain at the 3’ end (CD+) into EVs from HEK293T cells were compared. 50ng of pCD63-L7ae were co-transfected with indicated 50ng asRdRP Cat 800 in HEK293Tcells for 24h before supernatant were collected to detect asRdRP Cat 800 RNA relative to U6sRNA (housekeeping gene) by RT-PCR. Bars represent an average of three technical replicates ± SEM.


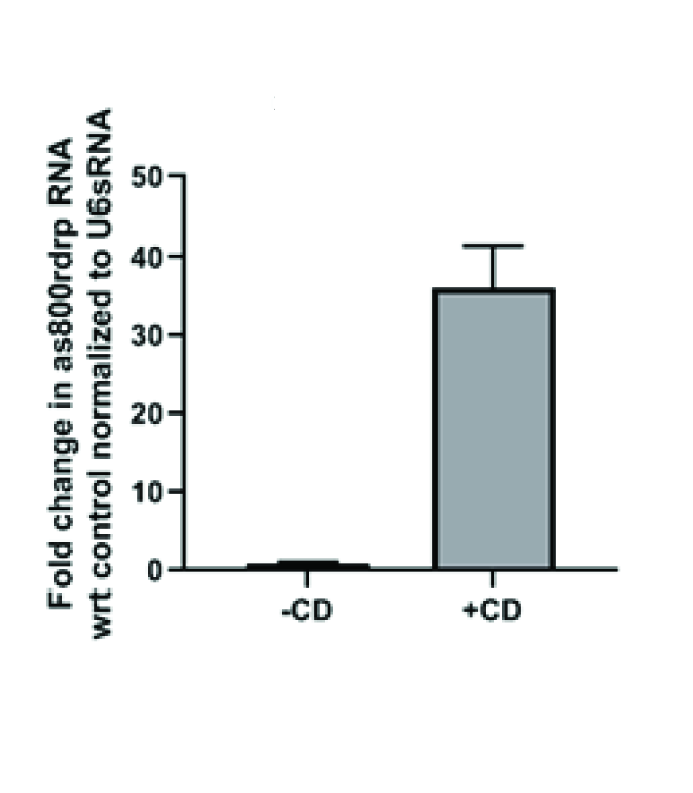

Supplement: Supplementary Figure S3 [file nat.2023.0078_suppl_figures3.docx]
