# Supplementary material for: Extracellular Vesicles Loaded with Long Antisense RNAs Repress Severe Acute Respiratory Syndrome Coronavirus 2 Infection
Source: Nucleic Acid Ther. 2024 Jun 17;34(3):101–8. doi: 10.1089/nat.2023.0078 (PMC11296208; doi:10.1089/nat.2023.0078)

**Figure S4** 100 billion HEK derived EVs or 30 billion NSC derived EVs were injected via IV by tail vein injection into C57BL/6 mice. PBS was used as control. Mice bled 72h post-IV before measuring liver enzyme panels. Each data point represents one mouse and error bars represent the SEM.


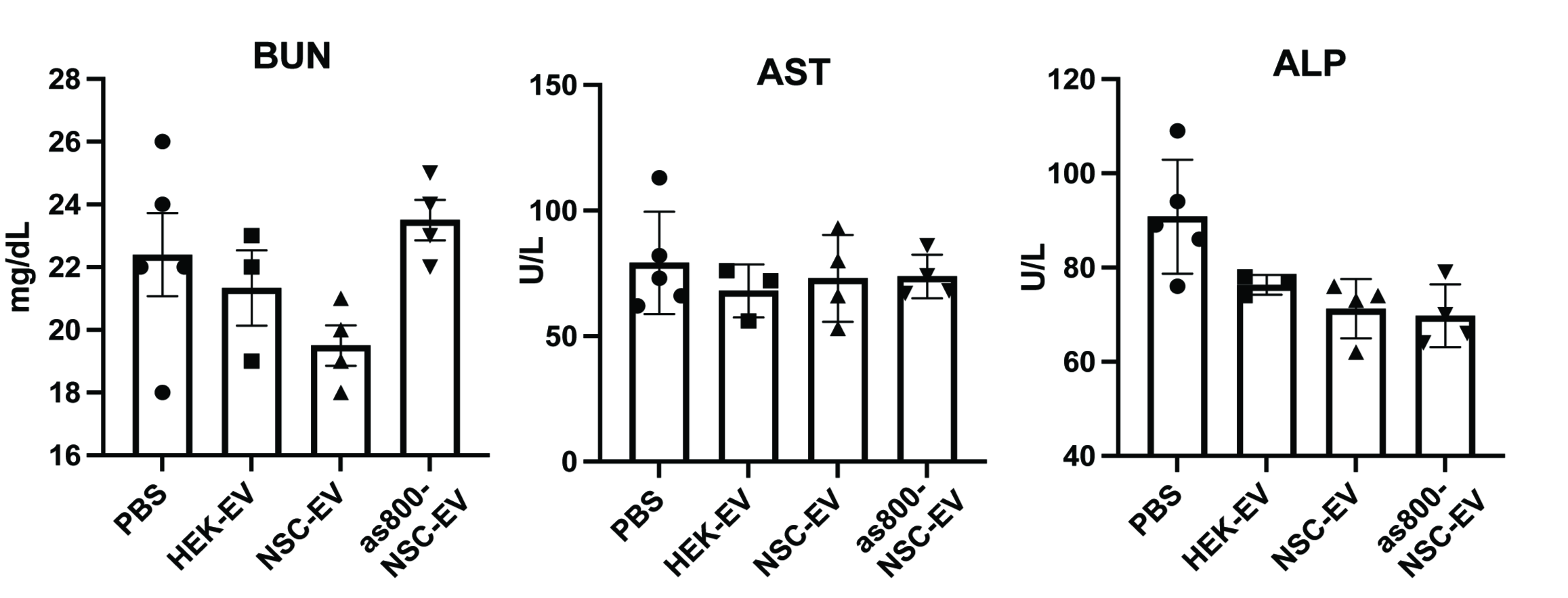

Supplement: Supplementary Figure S4 [file nat.2023.0078_suppl_figures4.docx]
