# Supplementary material for: Extracellular Vesicles Loaded with Long Antisense RNAs Repress Severe Acute Respiratory Syndrome Coronavirus 2 Infection
Source: Nucleic Acid Ther. 2024 Jun 17;34(3):101–8. doi: 10.1089/nat.2023.0078 (PMC11296208; doi:10.1089/nat.2023.0078)

**Figure S5** 100 billion HEK derived EVs or 30 billion NSC derived EVs were injected via IV by tail vein injection into C57BL/6 mice. PBS was used as control. Mice bled at 4 and 72h post injection to collect serum to measure cytokine expression by RT PCR. Bar represents mean and error bars represent the SEM (n = 3 to 5 mice).


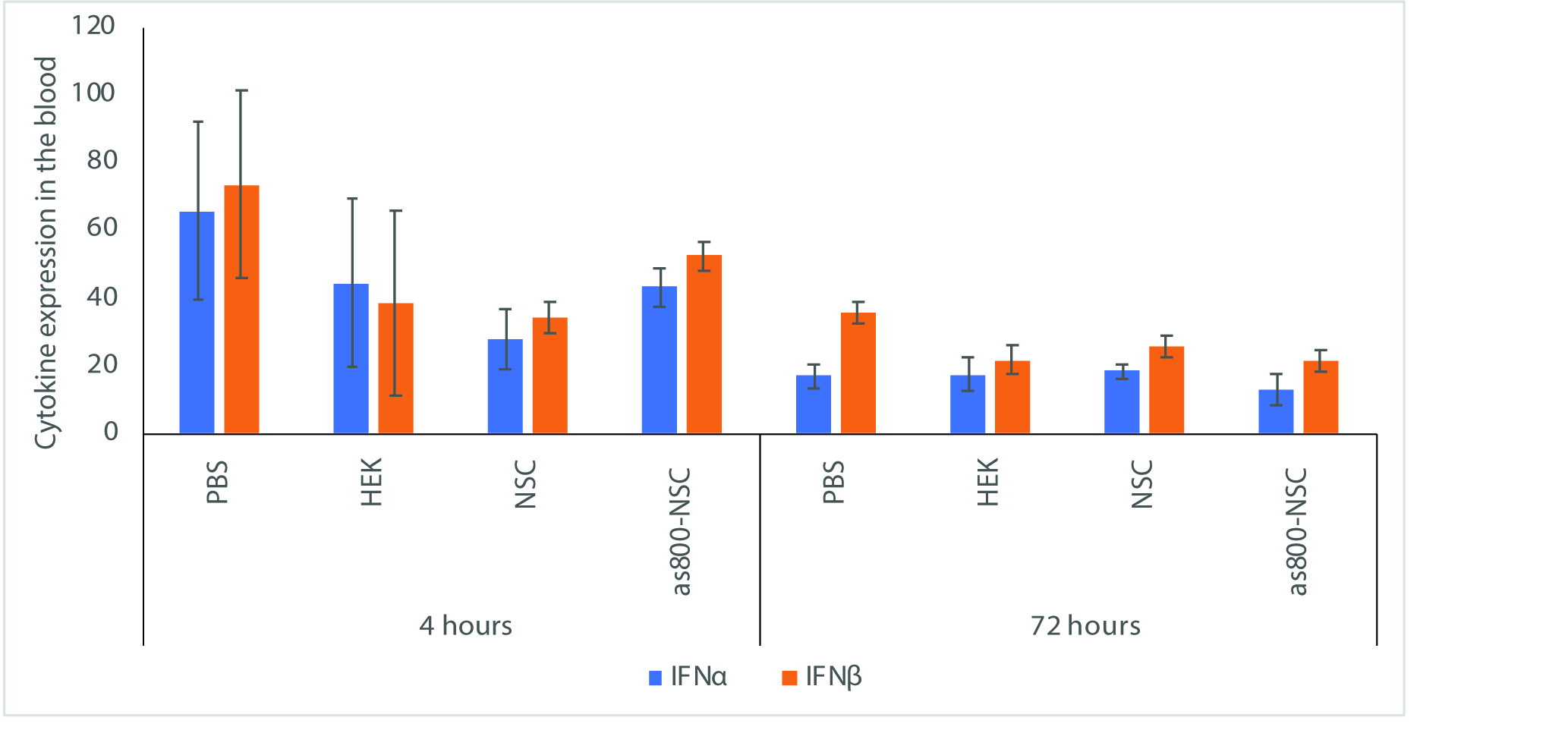

Supplement: Supplementary Figure S5 [file nat.2023.0078_suppl_figures5.docx]
