# Supplementary material for: Extracellular Vesicles Loaded with Long Antisense RNAs Repress Severe Acute Respiratory Syndrome Coronavirus 2 Infection
Source: Nucleic Acid Ther. 2024 Jun 17;34(3):101–8. doi: 10.1089/nat.2023.0078 (PMC11296208; doi:10.1089/nat.2023.0078)

**Figure S6** 7–13-week-old K18-hACE2 mice were intranasally (IN) infected with 10^4^ PFU of SARS-CoV-2. Mice were IV-administered with 30 billion NSC EVs in 100 µL of PBS by retro-orbital injection at 1, 2-, 3-, 4- and 5-days post-infection (dpi). At 5-7 dpi mice were euthanized, lung tissues were harvested and homogenized for RNA extraction. RT PCR for mouse IFNβ was performed relative to mouse GAPDH (housekeeping gene) for three independent mice per treatment group. Bars represent mean gene expression of three technical replicates and error bars represent SEM.


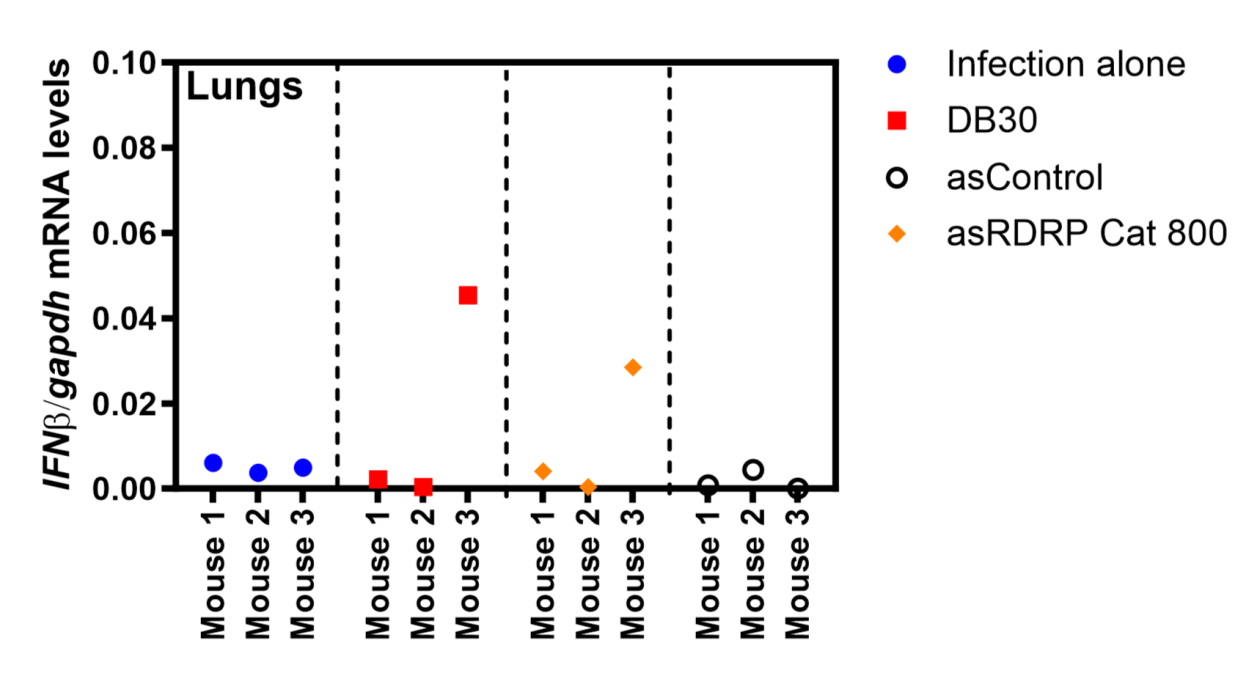

Supplement: Supplementary Figure S6 [file nat.2023.0078_suppl_figures6.docx]
