# Supplementary material for: Extracellular Vesicles Loaded with Long Antisense RNAs Repress Severe Acute Respiratory Syndrome Coronavirus 2 Infection
Source: Nucleic Acid Ther. 2024 Jun 17;34(3):101–8. doi: 10.1089/nat.2023.0078 (PMC11296208; doi:10.1089/nat.2023.0078)
Supplement: Supplementary Table S1 [file nat.2023.0078_suppl_tables1.docx]

**Supplementary table 1** Long asRNAs used in the current study were cloned in a manner to be expressed from the CMV promoter.

| eGFP as600 | tgccgtcctccttgaagtcgatgcccttcagctcgatgcggttcaccagggtgtcgccctcgaacttcacctcggcgcgggtcttgtagttgccgtcgtccttgaagaagatggtgcgctcctggacgtagccttcgggcatggcggacttgaagaagtcgtgctgcttcatgtggtcggggtagcggctgaagcactgcacgccgtaggtcagggtggtcacgagggtgggccagggcacgggcagcttgccggtggtgcagatgaacttcagggtcagcttgccgtaggtggcatcgccctcgccctcgccggacacgctgaacttgtggccgtttacgtcgccgtccagctcgaccaggatgggcaccaccccggtgaacagctcctcgcccttgct |
| --- | --- |
| eGFP as800 | caggtagtggttgtcgggcagcagcacggggccgtcgccgatgggggtgttctgctggtagtggtcggcgagctgcacgctgccgtcctcgatgttgtggcggatcttgaagttcaccttgatgccgttcttctgcttgtcggccatgatatagacgttgtggctgttgtagttgtactccagcttgtgccccaggatgttgccgtcctccttgaagtcgatgcccttcagctcgatgcggttcaccagggtgtcgccctcgaacttcacctcggcgcgggtcttgtagttgccgtcgtccttgaagaagatggtgcgctcctggacgtagccttcgggcatggcggacttgaagaagtcgtgctgcttcatgtggtcggggtagcggctgaagcactgcacgccgtaggtcagggtggtcacgagggtgggccagggcacgggcagcttgccggtggtgcagatgaacttcagggtcagcttgccgtaggtggcatcgccctcgccctcgccggacacgctgaacttgtggccgtttacgtcgccgtccagctcgaccaggatgggcaccaccccggtgaacagctcctcgcccttgct |
| N as600 | cttgtctgattagttcctggtccccaaaatttccttgggtttgttctggaccacgtctgccgaaagcttgtgttacattgtatgctttagtggcagtacgtttttgccgaggcttcttagaagcctcagcagcagatttcttagtgacagtttggccttgttgttgttggcctttaccagacattttgctctcaagctggttcaatctgtcaagcagcagcaaagcaagagcagcatcaccgccattgccagccattctagcaggagaagttcccctactgctgcctggagttgaatttcttgaactgttgcgactacgtgatgaggaacgagaagaggcttgactgccgcctctgctcccttctgcgtagaagccttttggcaatgttgttccttgaggaagttgtagcacgattgcagcattgttagcaggattgcgggtgccaatgtgatcttttggtgtattcaaggctccctcagttgcaacccatatgatgccgtctttgttagcaccatagggaagtccagcttctggcccagttcctaggtagtagaaataccatcttggactgagatctttcattttaccgtcaccaccac |
| N as800 | tttgtatgcgtcaatatgcttattcagcaaaatgacttgatctttgaaatttggatctttgtcatccaatttgatggcacctgtgtaggtcaaccacgttcccgaaggtgtgacttccatgccaatgcgcgacattccgaagaacgctgaagcgctgggggcaaattgtgcaatttgcggccaatgtttgtaatcagttccttgtctgattagttcctggtccccaaaatttccttgggtttgttctggaccacgtctgccgaaagcttgtgttacattgtatgctttagtggcagtacgtttttgccgaggcttcttagaagcctcagcagcagatttcttagtgacagtttggccttgttgttgttggcctttaccagacattttgctctcaagctggttcaatctgtcaagcagcagcaaagcaagagcagcatcaccgccattgccagccattctagcaggagaagttcccctactgctgcctggagttgaatttcttgaactgttgcgactacgtgatgaggaacgagaagaggcttgactgccgcctctgctcccttctgcgtagaagccttttggcaatgttgttccttgaggaagttgtagcacgattgcagcattgttagcaggattgcgggtgccaatgtgatcttttggtgtattcaaggctccctcagttgcaacccatatgatgccgtctttgttagcaccatagggaagtccagcttctggcccagttcctaggtagtagaaataccatcttggactgagatctttcattttaccgtcaccaccac |
| RdRP 5’ as600 | agaattgtactgtttttaacaaagcttggcgtacacgttcacctaagttggcgtatacgcgtaatatatctgggttttctacaaaatcataccagtcctttttattgaaataatcatcatcacaacaattgtatgtgacaagtatttcttttaatgtgtcacaattaccttcatcaaaatgccttaaagcatagacgaggtctgccattgtgtatttagtaagacgttgacgtgatatatgtggtaccatgtcaccgtctattctaaacttaaagaagtcatgtttagcaacagctggacaatccttaagtaaattataaattgtttcttcatgttggtagttagagaaagtgtgtctcttaactacaaagtaagaatcaattaaattgtcatcttcgtccttttcttggaagcgacaacaattagtttttaggaatttagcaaaaccagctactttatcattgtagatgtcaaaagccctgtatacgacatcagtactagtgcctgtgccgcacggtgtaagacgggctgcacttacaccgcaaacccgtttaaaaacgattgtgcatcagctgactgaagcatgggttcgcggagttg |
| RdRP 5’ as800 | agtgtcaacatgtgactctgcagttaaagccctggtcaaggttaatataggcattaacaatgaataataagaatctacaacaggaactccactacctggcgtggtttgtatgaaatcaccgaaatcataccagttaccattgagatcttgattatctaatgtcagtacaccaacaataccagcatttcgcatggcatcacagaattgtactgtttttaacaaagcttggcgtacacgttcacctaagttggcgtatacgcgtaatatatctgggttttctacaaaatcataccagtcctttttattgaaataatcatcatcacaacaattgtatgtgacaagtatttcttttaatgtgtcacaattaccttcatcaaaatgccttaaagcatagacgaggtctgccattgtgtatttagtaagacgttgacgtgatatatgtggtaccatgtcaccgtctattctaaacttaaagaagtcatgtttagcaacagctggacaatccttaagtaaattataaattgtttcttcatgttggtagttagagaaagtgtgtctcttaactacaaagtaagaatcaattaaattgtcatcttcgtccttttcttggaagcgacaacaattagtttttaggaatttagcaaaaccagctactttatcattgtagatgtcaaaagccctgtatacgacatcagtactagtgcctgtgccgcacggtgtaagacgggctgcacttacaccgcaaacccgtttaaaaacgattgtgcatcagctgactgaagcatgggttcgcggagttg |
| RdRP Cat as600 | taaagaactgacttaaagttctttatgctagccactagaccttgagatgcataagtgctattgaaacacacaacagcatcgtcagagagtatcatcattgagaaatgtttacgcaaatatgcgtaaaactcattcacaaagtctgtgtcaacatctctatttctatagagacactcataaagtctgtgttgtaaattgcggacatacttatcggcaattttgttaccatcagtagataaaagtgcattaacattggccgtgacagcttgacaaatgttaaaaacactattagcataagcagttgtggcatctcctgatgaggttccacctggtttaacatatagtgaaccgccacacatgaccatttcactcaatacttgagcacactcattagctaatctatagaaacggtgtgacaagctacaacacgttgtatgtttgcgagcaagaacaagtgaggccataattctaagcatgttaggcatggctctatcacatttaggataatcccaacccataaggtgagggttttctacatcactataaacagtttttaacatgttgtgccaaccaccatagaatttgcttgttccaattact |
| RdRp Cat as800 | taaagaactgacttaaagttctttatgctagccactagaccttgagatgcataagtgctattgaaacacacaacagcatcgtcagagagtatcatcattgagaaatgtttacgcaaatatgcgtaaaactcattcacaaagtctgtgtcaacatctctatttctatagagacactcataaagtctgtgttgtaaattgcggacatacttatcggcaattttgttaccatcagtagataaaagtgcattaacattggccgtgacagcttgacaaatgttaaaaacactattagcataagcagttgtggcatctcctgatgaggttccacctggtttaacatatagtgaaccgccacacatgaccatttcactcaatacttgagcacactcattagctaatctatagaaacggtgtgacaagctacaacacgttgtatgtttgcgagcaagaacaagtgaggccataattctaagcatgttaggcatggctctatcacatttaggataatcccaacccataaggtgagggttttctacatcactataaacagtttttaacatgttgtgccaaccaccatagaatttgcttgttccaattactacagtagctcctctagtggcggctattgatttcaataatttttgatgaaactgtctattggtcatagtactacagatagagacaccagctacggtgcgagctctattctttgcactaatggcatacttaagattcatttgagttatagtagggatgacattacgttttgtatatgcgaaaagtgcatcttgatcctcata |
